# Instability Resistance Training improves Working Memory, Processing Speed and Response Inhibition in Healthy Older Adults: A Double-Blinded Randomised Controlled Trial

**DOI:** 10.1038/s41598-020-59105-0

**Published:** 2020-02-13

**Authors:** Nils Eckardt, Claudia Braun, Armin Kibele

**Affiliations:** 1 University of Kassel, Institute of Sports and Sport Science, Department of Training and Movement Science, Kassel, 34121 Germany; 2 Carl von Ossietzky University of Oldenburg, Institute of Sport Science, Department of Sport and Movement Science, Oldenburg, 26129 Germany

**Keywords:** Ageing, Human behaviour, Randomized controlled trials

## Abstract

Aging is associated with declines in physical and cognitive performance. While there is no doubt about beneficial effects of physical exercise on proxies of strength and balance, the overall evidence for positive effects of resistance and balance training on executive functions is rather inconsistent. Whether the simultaneous exercising of strength and balance, i.e., instability resistance training, promotes executive functions in older adults is unknown. In the present trial, we tested the effects of unstable vs. stable resistance training on executive functions. Sixty-eight healthy older adults aged 65–79 years were randomly assigned to either an instability free-weight resistance training or one of two stable machine-based resistance training programs. Each group exercised twice a week on non-consecutive days for 10 weeks. Four tests to evaluate specific domains of executive functions were administered prior and following training: working memory, processing speed, response inhibition and set-shifting. The instability resistance training group improved working memory, processing speed and response inhibition from pre to post-test. In contrast, we found no improvements in executive functions for both stable resistance training groups. Our results demonstrate that 10 weeks of instability resistance training suffice to improve executive functions in older adults.

## Introduction

The decline of neuromuscular control, motor performance and cognition with aging and adverse health outcomes such as functional limitations and possible falls are major health care issues of the 21^st^ century^1–3^. Impaired executive functioning, as part of cognition, has been associated with reduced physical functioning and impaired locomotion in particular^3^. Therefore, improving executive functions and/or slowing age-related decline, is of great interest. While, based on a recent Cochrane review, the evidence for computerised cognitive training is somewhat inconclusive^4^, physical exercise interventions appear to be beneficial for executive functions based on single studies^5–11^. However, the overall evidence for beneficial effects of physical exercise interventions on executive functions is rather inconsistent^12,13^. Accordingly, we aimed to systematically investigate challenging and cognitively demanding vs. less demanding physical exercises^11–13^. Consequently, the effects of resistance training modalities with different cognitive and physical demands on executive functions in older adults were tested in this study.

The conflicting findings can mainly be explained by the abundance of different cognitive outcomes, tests and the variety of different study designs^6^ making comparisons difficult. Furthermore, Diamond and Ling^12,13^ argue that the reason why simple “mindless” exercise interventions like aerobic training (e.g., running on a treadmill) or pure balance training have little or no effect on executive functions is that they lack any cognitive challenge, attention or social component. Studies employing resistance training show that it may improve cognition in older adults^6,8,9^, yet consistent evidence of positive effects are still amiss^6,12,13^. All in all, it appears, that resistance training is more beneficial when it is challenging (e.g., progressive increase of load and sets)^6,9^. Similar to aerobic training, the abundance of cognitive outcomes, tests and exercise designs of resistance training interventions makes it difficult to compare existing study outcomes.

Unlike aerobic and resistance training, balance training is a less frequently practised exercise modality. However, in contrast to aerobic and resistance training, demanding balance training shows more consistent positive effects on executive functions^5,14^. We have to distinguish demanding balance training from ‘simple balance exercises’ or toning routines. The latter often require only one-legged balance tasks with displacements in the transversal plane and no displacement in the sagittal and frontal plane. In contrast, demanding balance exercises may include challenging eye–hand coordination, leg–arm coordination as well as spatial orientation and reaction demands to moving objects or persons in all planes^14^. It may as well include responses to perturbations, forcing participants to permanently re-stabilise within a metastable state of equilibrium^5,15^. It is not surprising that physical exercise interventions incorporating several training modalities and, therefore, providing higher demands and challenges, procure a superiority to basic aerobic and resistance training with regard to more consistent effects^6,7,16^. A recent randomised controlled trial (RCT)^17^ showed the feasibility and effectiveness of a multicomponent training, so called instability resistance training or resistance training on unstable surfaces (concurrent balance and resistance training), on proxies of strength, power and balance in older adults. Participants of the instability resistance group increased measures of balance, strength and power similar to the stable machine-based resistance training. Importantly, the instability resistance training group exercised with less than half the load during the major squat exercise (e.g., 52 kg vs. 20 kg). However, the trial did not test for cognitive effects. We know from previous research that both, resistance^6,8,9^ and balance^5,14^ training, may benefit cognition in older adults, particularly when it is challenging, raising the question whether the combination of both, i.e. balance and resistance training may enhance cognition in older adults, given the higher challenges and the need of increased attention.

Therefore, the present RCT aimed to determine whether physically and mentally challenging instability free-weight resistance training (I-FRT) affects cognitive performance in healthy older adults differently compared to traditional “less cognitive challenging” stable machine-based resistance training (S-MRT & S-MRT_HIP_). To assure, that the modality (unstable/stable) is the decisive factor, we implemented two stable groups to exclude any potential effect of a particular stable training program.

We hypothesised that the combined challenge of resistance training and balance training (instability resistance training) would result in increased executive functions after 10 weeks in comparison to stable resistance training. Secondly, we hypothesised that there would be no difference between the two different stable training modalities.

## Results

The exercise compliance for all participants over 10 weeks was on average 95.3 ± 0.86%. Demographic and baseline descriptors of the 68 participants who completed the 10-week trial are presented in Table 1. Baseline values at pre-test showed no differences between groups with respect to all investigated cognitive outcome variables (*ps* ≥ 0.122). Three participants were not able to conduct the Stroop-Colour-Word Test, due to colour-blindness. Furthermore, because of technical issues, post-test results for the Stroop-Colour-Word Test for one participant were not recorded. We found violations of normal distribution and homogeneity within the Trail Making Test and the Digit Symbol Substitution Test. The analysis with non-parametric tests revealed no changes in the outcomes. Accordingly, we only reported parametric results.

**Table 1:** Demographics and characteristics of participants at baseline.

Characteristics	Unstable		Stable				Baseline difference
	I-FRT (n = 21)		S-MRT (n = 24)		S-MRT_HIP_ (n = 23)		
	*M*	*SD*	*M*	*SD*	*M*	*SD*	*p-value*
Age (years)	71.3	3.9	69.5	3.8	69.9	3.9	0.288
Body height (cm)	171	9	169	7	1.69	9	0.820
Body mass (kg)	76.9	15.7	73.8	12.4	76.6	13.6	0.691
Sex (f/m)	12/9		16/8		13/10		−
Physical activity (h/w)	9.4	9.2	11.9	8.6	12.2	7.2	0.215*
MMSE	27.9	1.6	27.8	1.8	28.0	1.6	0.914*
CDT	all participants were classified as non-pathological						
GDS	0.9	1.0	1.1	1.6	1.0	1.4	0.957*
FAB_D	15.1	2.1	15.3	2.0	15.7	2.2	0.521*

*Note*: I-FRT = instability free-weight resistance training, S-MRT = stable machine-based resistance training, S-MRT_HIP_ = stable machine-based adductor/abductor training; M = mean; SD = standard deviation; f = female; m = male; MMSE = Mini Mental State Examination; CDT = Clock Drawing Test; GDS = Geriatric Depression Scale; FAB_D = Frontal Assessment Battery, German Version. P-values denoted with an * violated distribution assumptions and non-parametric differ from parametric results and are displayed.

All outcome measures are displayed in Table 2.

**Table 2:** Outcomes of the exercise intervention for pre- and post-testing.

Variables	Unstable				Stable							
	I-FRT (*n* = 21)				S-MRT (*n* = 24)				S-MRT_HIP_ (*n* = 23)			
	pre		post		pre		post		pre		post	
	*M*	*SD*	*M*	*SD*	*M*	*SD*	*M*	*SD*	*M*	*SD*	*M*	*SD*
**DSST**												
Number	37.8	8.11	45.0	8.2	42.9	8.2	44.7	8.5	40.9	8.1	42.9	9.7
**DMT**												
Score	97.0	14.9	107.3	14.9	99.7	13.0	99.8	16.4	98.4	11.3	98.3	13.2
**Stroop**												
Circle (ms)	623.3	109.4	620.3	115.0	602.7	91.5	622.3	69.8	629.9	82.6	631.1	69.4
Neural (ms)	754.9	124.4	714.5	125.6	706.0	142.6	718.8	104.7	757.5	98.9	721.3	94.1
Incongruent (ms)	903.2	146.6	829.2	139.1	863.1	167.7	862.5	144.4	886.6	126.2	885.8	138.12
Score	1.45	0.14	1.35	0.12	1.43	0.18	1.39	0.18	1.41	0.19	1.40	0.14
**TMT**												
A (s)	41.9	16.6	31.6	8.2	36.6	9.4	31.4	7.7	42.0	12.3	31.5	10.6
B (s)	96.0	42.9	76.2	30.1	95.7	42.6	81.0	51.9	108.1	43.0	80.9	33.9
Ratio	54.1	32.8	44.7	24.2	59.1	38.7	49.6	48.7	66.1	40.1	46.5	35.0

*Note*: I-FRT = instability free-weight resistance training, S-MRT = stable machine-based resistance training, S-MRT_HIP_ = stable machine-based adductor/abductor training; *M* = mean; *SD* = standard deviation. DSST = Digit Symbol Substitution Test; DMT = Digit Memory Test; Stroop = Stroop Colour-Word Test; TMT = Trail Making Test.

### Falls efficacy scale

The fear of falling was overall reduced by 3–7%. However, there were no differences between groups.

### Executive functions

#### Digit memory test

**Figure 1:**
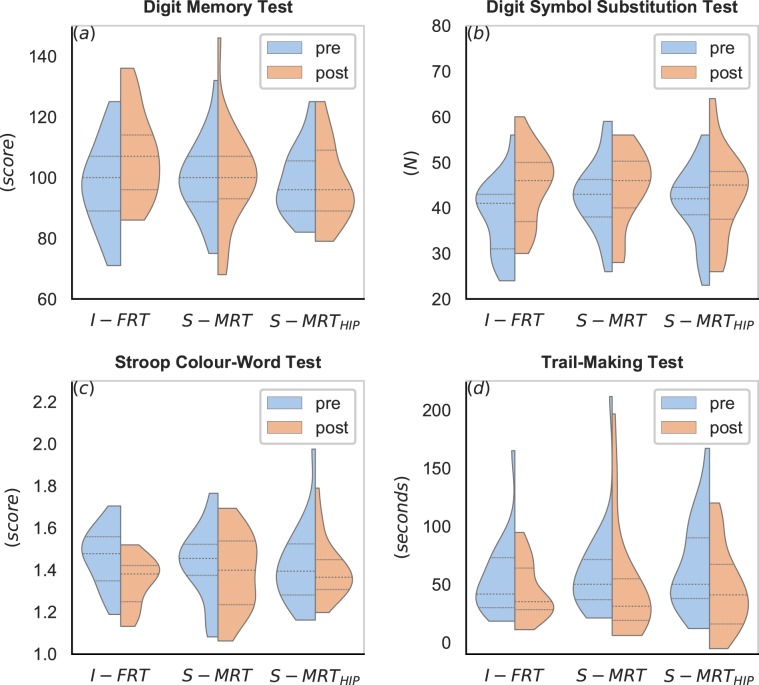
Violinplots showing the results for the executive functions pre (blue) and post (orange) intervention. I-FRT = instability free-weight resistance training, S-MRT = stable machine-based resistance training, S-MRT_HIP_ = stable machine-based adductor/abductor training. dashed line = median; dotted line = upper/lower quartile. The width of the plots is scaled to data distribution.

There was a significant effect between groups (*d* = 0.32). The planned contrasts revealed that the condition “unstable” improved working memory significantly by 11% compared to the “stable” condition (*d* = 0.32), which did not change at all (0%). See Fig. 1a and Table 3.

**Table 3:** Statistical results. *Note*: I-FRT = instability free-weight resistance training, S-MRT = stable machine-based resistance training, S-MRT_HIP_ = stable machine-based adductor/abductor training; FES-I = Fall Efficacy Scale. DSST = Digit Symbol Substitution Test; DMT = Digit Memory Test; Stroop = Stroop Colour-Word Test; TMT = Trail Making Test; TuT = Time under Tension; *no pre-post measures, thus only *t*-tests were calculated; BF = Bayes Factor; p ≤ 0.05.

	ANOVA					planned contrasts				95%*-CI (d*_*unb*_)
	*df*	*F*	*p*	*d*		*df*	*t*	*p*	*d*_*unb*_	
FES-I	2,65	0.84	0.436	0.32						
					Unstable vs. Stable	65	0.92	0.364	−0.24	−0.75, 0.28
					S-MRT vs. S-MRT_HIP_	65	0.90	0.373	0.27	−0.30, 0.85
DSST	2,65	3.95	0.024	0.53						
					Unstable vs. Stable	65	2.81	0.007	0.73	0.21, 1.27
					S-MRT vs. S-MRT_HIP_	65	0.08	0.937	−0.02	−0.60, 0.55
DMT	2,65	6.05	0.004	0.66						
					Unstable vs. Stable	65	3.48	< 0.001	0.91	0.38, 1.45
					S-MRT vs. S-MRT_HIP_	65	0.09	0.928	0.02	−0.55, 0.60
Stroop	2,61	2.36	0.103	0.42						
					Unstable vs. Stable	61	2.09	0.041	0.55	0.01, 1.09
					S-MRT vs. S-MRT_HIP_	61	0.66	0.510	−0.20	−0.79, 0.40
TMT	2,65	0.94	0.394	0.34						
					Unstable vs. Stable	43	0.68	0.502	0.17	−0.34, 0.69
					S-MRT vs. S-MRT_HIP_	45	1.20	0.233	0.33	−0.24, 0.91
***t-tests***										
Load*					S-MRT vs. I-FRT	43	15.24	< 0.001	4.34	3.34, 5.46
					S-MRT vs. S-MRT_HIP_	45	9.19	< 0.001	2.64	1.88, 3.48
					I-FRT vs. S-MRT_HIP_	42	9.86	< 0.001	2.72	1.98, 3.52
TuT*					S-MRT vs. I-FRT	43	2.98	0.006	1.11	0.32, 1.96
					S-MRT vs. S-MRT_HIP_	45	6.29	< 0.001	2.31	3.35, 1.39
					I-FRT vs. S-MRT_HIP_	42	9.05	< 0.001	3.27	4.50, 2.20

#### Digit symbol substitution test

We found a medium effect between groups (*d* = 0.53). While I-FRT improved by 19%, the “stable” modality improved only by 4.5%. The effect is also supported by the planned contrast analysis, revealing a medium effect (*d* = 0.73) in favour of the “unstable” condition compared to the “stable” condition. See Fig. 1b and Table 3.

#### Stroop-colour-word test

There was no significant effect of the pre-post difference between groups (*d* = 0.42). However, we found a medium effect (*d* = 0.55), when comparing the “unstable” condition with the “stable” condition in favour of higher pre-post differences for I-FRT (8% for I-FRT and less than 3% for the stable groups) (see Fig. 1c and Table 3). Individual errors, like uttering a wrong colour, were rare (<5%) and evenly distributed across pre- and post-testing and groups. Therefore, errors were not used for any further analysis.

#### Trail making test

The analysis for the Trail Making Test remained non-significant, the ANOVA (*d* = 0.34) and the planned contrasts (*d* = 0.17) revealed only small effects, indicating no difference between groups (see Fig. 1d and Table 3).

### Training intensity

Given the inter-individual differences of the participants, load and TuT were not normally distributed, therefore we used non-parametric statistics.

#### Load

**Figure 2:**
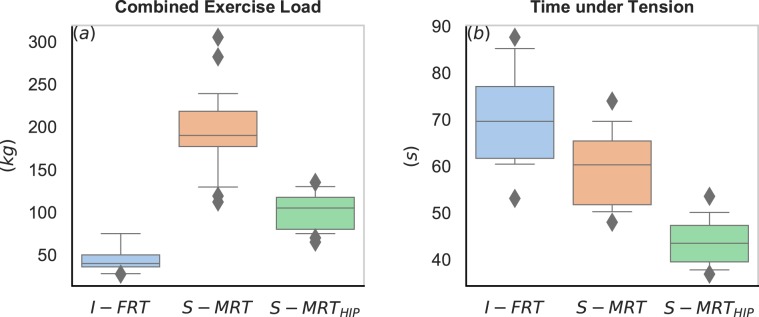
Boxplots showing the maximal training load (**a**) and Time under Tension (**b**) during the last training phase. I-FRT = free-weight instability resistance training (blue), S-MRT = machine based stable resistance training (orange), S-MRT_HIP_ = machine-based adductor/abductor training (green). The box shows the quartiles of the dataset while the whiskers extend to show the rest of the distribution. Outliers are plotted as individual diamond shaped points.

On average, I-FRT exercised in total with ~150 kg less than S-MRT and with ~56 kg less than S-MRT_HIP_, implicating that I-FRT exercised with considerably lower loads than the other groups. The numeric differences were reflected in the statistical analysis revealing very large effects (*d* ≥ 2.72) for higher loads of the stable modalities (see Fig. 2a and Table 3).

#### Time under Tension (TuT)

The *t*-tests revealed that I-FRT exhibited greater overall TuT in comparison to S-MRT (~13 s) and even greater TuT compared to S-MRT_HIP_ (~26 s) per set across both main exercises (see Fig. 2b and Table 3).

## Discussion

The goal of the present three-arm double-blinded RCT was to compare the effects of instability resistance training vs. stable resistance training on executive functions in older adults. Executive functions were assessed by performing four established tests: the Digit Memory Test, the Digit Symbol Substitution Test, a computerised Stroop-Colour-Word Test (Victoria Version) and the Trail Making Test. We first hypothesised that a multicomponent challenging exercise modality (i.e., instability resistance training) would exceed the effects of the less challenging stable machine-based resistance training in healthy older adults. In agreement with our first hypothesis we found meaningful improvements from pre- to post-testing for I-FRT compared to the stable resistance training groups in the Digit Memory Test, the Digit Symbol Substitution Test and the Stroop-Colour-Word Test. We detected similar improvements across all groups in the Trail Making Test, with no particular advantage for one group or modality. Our second hypothesis, that there would be no difference between stable training modalities was confirmed by the planned contrast analysis, indicating that the feature “unstable”, which imposes higher challenges, physically as well as mentally, might play the pivotal role in improving executive functions in healthy older adults. The overall outcome of our RCT suggests that free-weight instability resistance training is capable to improve executive functions in healthy older adults within 10-weeks. These findings extend previous study results of multicomponent exercise interventions on executive functions in older adults^6,7^.

Our previous research advocates the feasibility and effectiveness of instability resistance training^17^. Feasibility (high compliance and no training-related drop outs) was confirmed by this study. The novelty of the current RCT compared to our last RCT^17^ is the effect on executive functioning, given that no tests of executive functions were administered the last time. In contrast to previous investigations^8,14^ showing that exercise interventions affected only selective domains, our results suggest that instability free-weight resistance training appears to affect all tested executive functions. Indeed, we found medium effects for an improvement in working memory, processing speed and response inhibition for the I-FRT group compared to the stable modalities, while all groups demonstrated improvements over time for the Trail Making Test. In comparison, previous research (e.g. Forte and colleagues^18^) found pre-test to post-test improvements of 29% in the Trail Making Test after four weeks of training while no meaningful increase was reached after three months. They argued that these changes were probably due to a learning effect. Accordingly, such a learning effect should be present in all non-exercising control groups. However, looking thoroughly at control groups in other studies, conflicting results can be detected^19–21^. While Klusmann and collegues^20^ stated a decline in the Trail Making Test B/A by 10% after six months of training, Napoli *et al*.^19^ found no change at all (0.8–1.7%) after one year of exercise intervention. Contrary to these results, Vaughan and colleagues^21^ found improvements in the Trail Making Test B within the control group by 7%, although the effect was small (*d* = 0.25). Furthermore, a recent review^6^ reported meaningful improvements in Trail Making Tests when comparing exercise interventions with passive controls. We used a modified version of the Trail Making Test with a different arrangement of numbers and letters for post-testing, therefore, we cannot exclude the possibility that improvements are due to an easier post-test. Thus, given the inconclusive study results in the Trail Making Test, we follow the argument by Forte and colleagues^18^ assuming pre- to post-differences to originate from a learning effect.

It seems that that the combined demands of resistance training and balance training promote and accelerate beneficial effects of physical activity on executive functions. The participants in this study exercised for 10-weeks. The duration of most studies covered longer periods of time (more details in the review by de Asteasu *et al*. 2017)^6^. Only a few RCTs lasted three to four months with mixed results. For example, while Forte and colleagues^18^ found positive effects in executive functioning for the resistance training group, Kimura *et al*.^22^ and Barnes *et al*.^23^ found no positive impact on cognition for this type of training. Notably, a resistance training program conducted by Liu-Ambrose and colleagues^8^ promoted positive effects on cognition after 12 but not after 6 months of intervention. A recent study by Rogge and colleagues^5^ found meaningful improvements in memory and spatial cognition after a 12 week balance training. However, no effects on executive functions were found. This might be due to the fact that the tested population consisted of young and healthy adults possibly not yet facing age-related negative declines of executive functioning. Therefore, younger participants may not be as susceptible to improvements through physical activity as older adults are.

Cortical plasticity^24,25^ and neurogenesis^26^ were identified as potential mechanisms which may increase cognitive performance in consequence of balance training. Increased production of neurotrophic growth factors like insulin growth factor 1 (IGF-1), brain-derived neurotrophic factor (BDNF) and reduced levels of homocysteine^27–29^ are assumed to play a role in preventing cognitive decline through resistance training. Indeed, increased levels of IGF-1 and BDNF seem to promote neural growth and improved cognitive performance^27,28^. Opposed to increased levels of IGF-1 and BDNF, increased levels of homocysteine are associated with impaired cognitive performance^29^ due to its neurotoxicity^30^. In addition, resistance training results in positive cortical adaptations similar to those of aerobic training^31^. Aerobic training can induce structural changes in the brain volume of older adults, more specifically in grey matter (anterior cingulate cortex, supplementary motor areas, posterior middle frontal gyrus and left superior temporal lobe) and in white matter (anterior third of corpus callosum)^32^. However, Voelcker-Rehage and colleagues^14^ recently pointed out that aerobic and coordination training improve cognitive performance in older adults in different ways. They found unchanged activity patterns in the superior, middle and medial frontal cortex as well as the superior and middle temporal cortical areas as a result of coordination training. Still, cognitive performance increased overall as compared to a control group. The authors argued that coordination training economises cognitive processes and facilitates automatization. Thus less compensatory activation is needed to counteract degenerative aging effects especially as higher prefrontal activation seems to be associated with age-related structural and functional decline^14^. The increased activation in untrained older adults may indicate compensatory mechanisms of executive control to counteract neural processing impairments^14,33^. Therefore, lower brain activation levels in line with exercise related improvements in cognitive performance would indicate a more efficient processing and automatization. In addition, Niemann and colleagues^34^ pointed out that balance and coordination training engages the (pre)frontal and parietal cortex and the basal ganglia in a similar way which can be associated with early stages of motor learning. While performing a balance task, we are faced with constant, yet unique challenges and subsequent adaptive processes comparable to early motor learning stages. Thus, balance appears to trigger neuroplasticity on multiple levels, structurally by increasing grey and white matter and by optimising signal processing and freeing cognitive resources. Interestingly, we found that instability resistance training affected motor signal processing by reducing motor noise during challenging locomotion^35^. The uncontrolled manifold control analysis (UCM) was used to calculate multijoint-covariation related to the stabilization of a particular performance variable. The UCM analysis tests the extent to which all available degrees of freedom (DoF) that contribute to a task-relevant performance variable co-vary so as to stabilize, i.e., reduce the variance of, that performance variable. Within the UCM analysis, variability is partitioned into two components: “good” variance that has no effect on the performance variable and “bad” variance, that results in a variable performance. The unstable environment provided by an instability resistance training apparently stimulates exploration of motor solutions in a manner that appears to transfer to challenging locomotor tasks. A recent consensus paper pointed out the cerebellum’s role in movement and cognition, particularly the interaction of prefrontal structures and the function of the cerebellum in movement automatization, as well as mediating executive functions^36^. Given the effect of instability resistance training as a tool to reduce the motor noise through a better state estimation within the anticipatory control-loop^35^, the here reported improvements in executive functions, and the possible structural and functional connections, it appears obvious that instability resistance training benefits cognitive processes which in turn may positively affect motor control.

Without doubt, instability resistance training is challenging, both physically and mentally. The continuous combination of movements to counteract perturbations, challenges the vestibular system^5^, cognition, as well as neuromuscular activation^37–39^. In fact, this combination of modalities appears to facilitate and augment complementary effects of physical exercise training on executive functions in older adults. Physical exercise promotes the release of neurotrophic factors such as IGF-1 and BDNF and thus may prepare the central nervous system to process the cognitive demands of instability resistance training more efficiently and enduring^40,41^. This is in line with an argument by Moreau and Conway (2013) stating that programs which are characterised by complexity, novelty and variety would be the most efficient at promoting executive functions^42^.

The reasons why we did not detect any meaningful effect within both stable machine-based resistance training groups may be versatile. On the one hand, the duration of our RCT was substantially shorter when compared to other RCTs in which significant effects were found^8^. Therefore, we cannot exclude the possibility that longer intervention periods may, in fact, provide positive effects on executive functions through stable machine-based resistance training. On the other hand, our participants were cognitively healthy older adults based on the Mini-Mental-State-Examination and the Frontal Assessment Battery. Thus, the higher effort of the additional balance challenges and/or longer TuT of the instability resistance training group may have been necessary to elicit changes within three months of intervention in this cognitively healthy population.

A limitation that warrants discussion is that our results cannot be generalized to less healthy or frail older adults. Even though, frail older adults are capable to adapt to physical exercise similar to healthy older adults^43^. Following the argumentation of Diamond and Ling^12^, attention, reduction of stress, and loneliness can be driving forces in improved executive functions in older adults. Given the blinding of the instructors and assessors, it appears unlikely that one group received more attention than the others. However, we cannot rule out that instability resistance training might have affected stress-reduction more than stable resistance training. In addition, it might be possible that participants of the I-FRT group bonded more than participants of the stable RT groups over the more challenging RT and mastering this challenge. Nonetheless, further investigation is needed. In addition, we can only speculate about neuroplasticity since we did not use any functional imaging techniques in our study. Furthermore, improvements of I-FRT might only be due to longer TuT, given that this parameter was not controlled for. However, there were no differences between both stable groups, although TuT differed considerably. Therefore, we’d suggest that TuT is not a decisive factor for improvements of unstable resistance training, nonetheless, this issue needs further investigation.

In conclusion, given the importance of cognition and, more specifically, executive functions with aging, this RCT provided evidence about the feasibility and effectiveness of multicomponent instability resistance training in older adults after 10 weeks of progressive training. This extends existing findings on exercise interventions to promote executive function in older adults. High exercise loads do not seem to be mandatory to induce improvements in older adults neither on a physical level^9,17^ nor a cognitive level as was shown here. Free-weight instability resistance training possibly affects the central nervous system on different organisational levels, ranging from neuroplasticity to neurotrophins. From an applied point of view, this seems reasonable, given that biological systems are highly interlinked and interdependent. Thus, we suggest that free-weight instability resistance training may not only serve as an alternative to traditional machine-based resistance training. The effects on executive functions and the instability-dependent decrease of the absolute training load make it especially suitable for those individuals restricted by high strain, like older adults.

## Methods

This investigation is part of the Kassel Fall Prevention Study II exploring the effects of three different resistance training modalities on locomotor control and proxies of strength and balance in older adults as stated within the trial registration. Due to the relevance of cognition in fall prevention research and the distinctive nature of the field of research, we decided to publish and discuss the results for the cognitive tasks separately. The effects on locomotor control and proxies of strength and balance are published and discussed elsewhere^35^. Sample size calculations where based on different outcome variables (see Eckardt & Rosenblatt (2019)^35^ trial registration).

### Study design

The study is part of a registered three-arm, double-blinded (assessors as well as participants were blinded) RCT (ClinicalTrials.gov: NCT03017365 on 01/04/2017) examining the effects of primarily unstable vs. unstable resistance training on executive functions in older adults. Participants were naïve to the study hypothesis. The local ethics committee of the University of Kassel gave their approval (E052016058) and we complied with the relevant ethical standards of the latest Declaration of Helsinki (WMA, Oct. 2013). All participants provided written informed consent prior to enrolment.

### Participants

**Figure 3:**
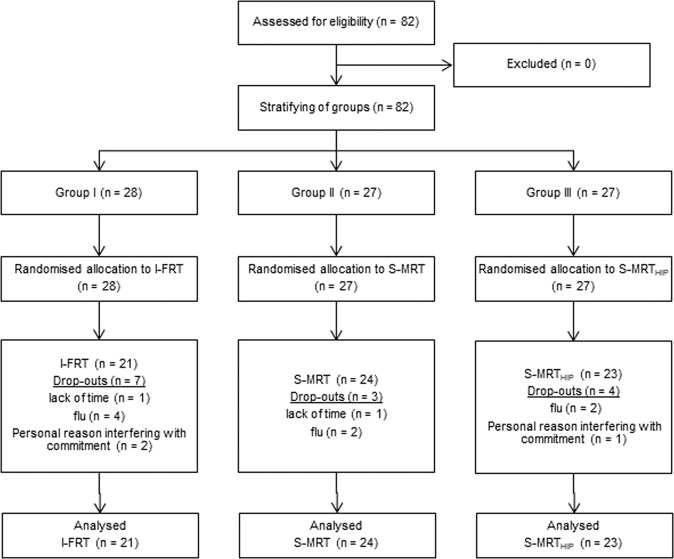
CONSORT diagram with participant flow.

We recruited 82 participants (range: 65–80 years) via public advertisement in a local newspaper. Inclusion criteria were determined as the ability to walk independently without any walking aid and normal or corrected-to-normal vision. To account for possible cognitive and mental health conditions, participants were excluded based on pathological ratings of the Clock Drawing Test (CDT)^44^, the Mini-Mental-State-Examination (MMSE,<24 points)^45^, the Falls Efficacy Scale – International (FES-I, >24 points)^46,47^, the Geriatric Depression Scale (GDS, >9 points)^48^, the Freiburg Questionnaire of Physical Activity (FQoPA, <1 h)^49^ and the Frontal Assessment Battery (FAB-D, <13 points)^50^. Sixty-eight participants completed the trial. Figure 3 shows the CONSORT flow diagram and the number of participants in the treatment arms at each stage of the trial.

### Randomisation

We stratified participants (1:1:1) into one of three groups based on age and sex. An independent assessor randomly assigned the groups to one of three training modalities: S-MRT, I-FRT, or S-MRT_HIP_. The randomisation sequence was generated using www.randomizer.org and concealed until groups were stratified.

### Assessments

Data was collected in the biomechanics laboratory of the University of Kassel, Germany. Four participants were tested simultaneously by different assessors assigned to specific tests, respectively. The allocation of participants to the assessors was carried out randomly.

Global cognitive functioning was assessed using the Mini-Mental-State-Examination, a screening tool for mild cognitive impairment^45^. The Frontal Assessment Battery consists of six neuropsychological tasks evaluating cognitive and behavioural frontal lobe functions^51^. Physical activity was assessed using the Freiburg Questionnaire of Physical Activity^49^. Individual concerns about falling were evaluated using the Falls Efficacy Scale International^46^.

A recent systematic review identified processing speed, memory, set-shifting and response inhibition as key domains of executive functions related to the risk of falls^52^. Therefore, we choose to assess these key domains by using the following tests:

#### Executive function assessment

To test working memory, we used the Digit Memory Test^53^. This test consists of two parts, A: digit forwards and B: digit backwards. The assessor read out aloud random sequences of numbers (one per second), beginning with three digits and ending with nine digits. Participants had to repeat each sequence exactly in the given order of numbers. Part A consisted of repeating the numbers in a forward direction and Part B in a reversed backward direction. The total number of correct responses backwards and forwards were added up and converted into a standard score^54^ as an index for working memory. We used a different arrangement of digits for the post test (e.g.; pre: 296; post: 548). The test-retest reliability for our age groups (65–80 years) is considered from acceptable to good (ICC’s 0.71–0.88)^53^.

We administered the Digit Symbol Substitution Test^55,56^, which requires response speed, sustained attention, visual spatial skills and set shifting. Participants were asked to fill in a series of symbols correctly coded within 120 seconds. The first seven digit-symbol combinations where used as test-trial and neither measured nor included in the analysis. We arranged a different combination of digit-symbols for the post test. The test-retest reliability for the Digit Symbol Substitution Test is acceptable to excellent, ranging from 0.79 to 0.97^56^.

To assess selective attention and conflict resolution, we administered a computerised Stroop-Colour-Word Test (Victoria Version)^57,58^. The stimuli (24 per condition, 3 conditions) were presented in a random order on a computer screen. Participants were asked to verbally utter the presented colour as fast as possible. During condition I, coloured circles in green, blue, yellow, or red were shown. In condition II, neutral but coloured words (loud, above, hard or strong [in German]) were presented. Lastly, condition III consisted of incongruently coloured words (green, blue, yellow or red [in German]). We used E-Prime 2.0 (Psychology Software Tools, Inc., Sharpsburg, USA) to control stimulus presentation and record reaction times. Reaction times <250 ms and >3*interquartile range were dismissed. The ability to selectively attend and control response output was calculated as the time ratio (i.e., Stroop score) of colour-word interference and colour only tasks (condition III / condition I). If the participant’s voice did not trigger the microphone or the participant made a noise other than a response directed at naming the colour (i.e., vocalised pause: “um,” “uh”), the experimenter coded an error. Wrong responses were also coded as an error. The test-retest reliability for the Stroop-Colour-Word Test was previously reported to be good (ICC’s 0.71–0.79)^57,59^. It is noteworthy that the reliability is based on the use of response times^59^.

We used the paper & pencil version of the Trail Making Test (A + B)^60,61^ to assess set shifting and processing speed. To complete Part A, participants had to draw a line from 1 to 2, 2 to 3, 3 to 4, etc. Part B included additional letters, so that participants had to draw a line from 1 to A, A to 2, 2 to B, B to 3, etc., while the numbers were printed spatially distributed across the sheet. The time (in seconds) to complete the task was recorded with an ordinary stop watch. To quantify set shifting, we calculated the difference between Part B and Part A. The lower the scores the better the set shifting ability^8^. We used a different arrangement of letters and numbers for the post test. The test-retest reliability of the Trail Making Test across alternate versions was reported to be from acceptable to good (ICC’s 0.76–0.89)^62^.

### Exercise intervention

All three groups began training one week after the baseline assessments were completed. Training was supervised by two skilled instructors at all times (participant-to-instructor ratio of 5:1). The instructors recorded attendance and compliance (percentage of the total classes attended) was calculated using the attendance records. All intervention groups trained for 10 weeks, twice per week on non-consecutive days for, at most, 60 min each. The 10-week intervention period consisted of a one-week introductory phase and three major training blocks lasting three weeks each. Training intensity was progressively and individually increased from block to block over the 10-week training programme by modulating load and sets for all groups and the level of instability for group I-FRT. After week one, four and seven the training load (weight) was increased following one repetition maximum (1-RM) testing using the prediction equation provided by Epley^63^ for each major exercise. The 1-RM was performed under stable conditions for every group.

### S-MRT

This group executed a) squats at the Smith machine and worked out at the b) leg-press. Secondary exercise were core exercises.

### I-FRT

The main exercises of this group were squats too, however they were conducted using free weights and instability devices. In addition, the participants of this groups conducted front-lunges on instability devices. The secondary exercises were core exercises incorporating instability devices.

### S-MRT_HIP_

**Figure 4:**
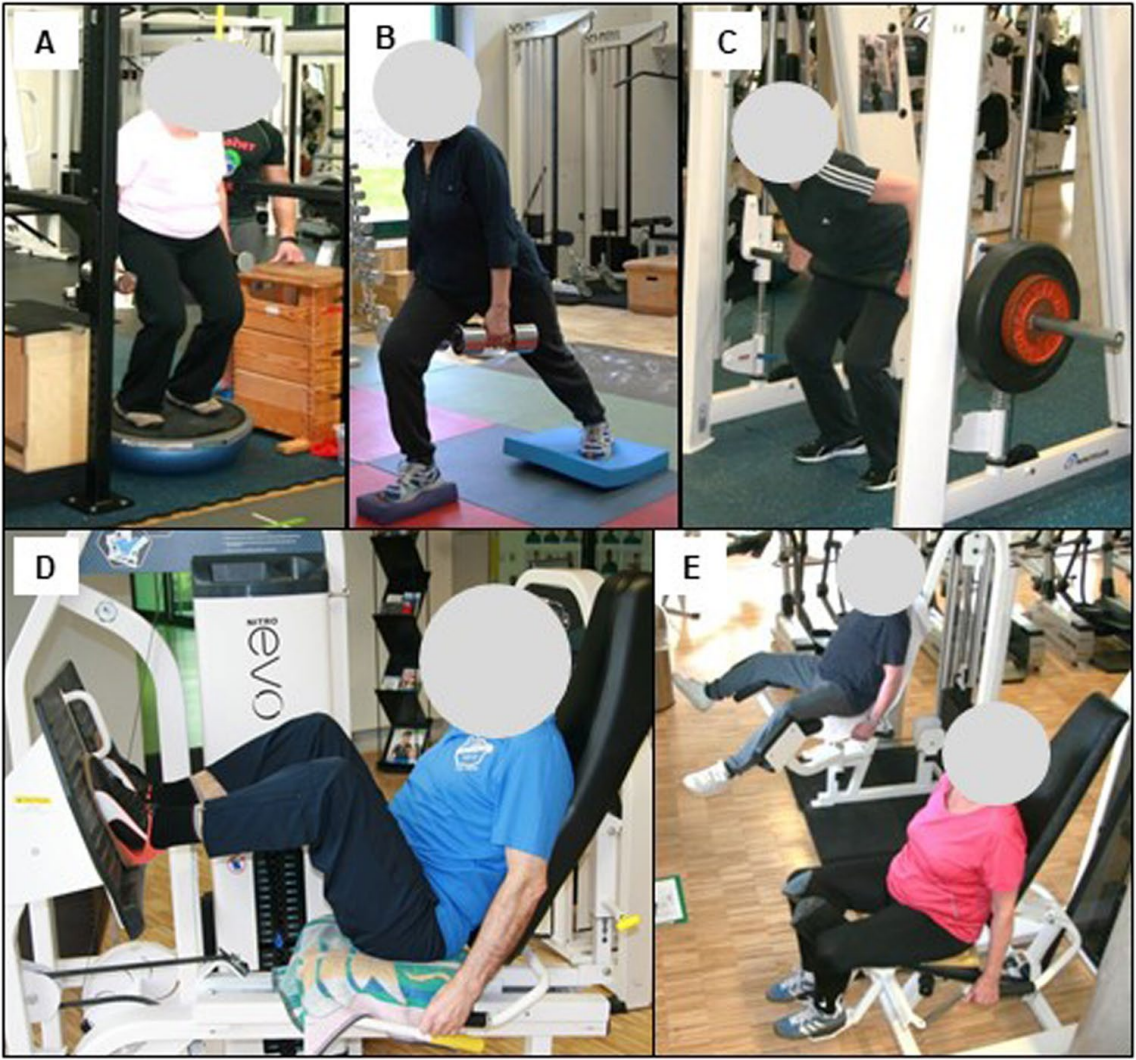
Photographs of the main exercises. (**A**) Squats using instability devices and dumbbells; (**B**) front lunges; (**C**) Squats at the Smith machine, placing the barbell at the hip, (**D**) Leg Press (**E**) thigh/hip adductor- and abductor resistance machine.

The focus of this training group were exercises targeting the thigh/hip adductors and abductors at resistance machines (see Fig. 4E). Secondary exercises were adduction and abduction exercises using elastic rubber straps. In addition, lateral core exercises were introduced.

A detailed description of the training programme, machines, equipment and progression is outlined in Table 4 and in Eckardt & Rosenblatt (2019)^35^.

**Table 4:** Detailed intervention program for all groups and phases. *Note:* I-FRT = free-weight instability resistance training; S-MRT = stable machine-based resistance training, S-MRT_HIP_ = stable machine-based adductor/abductor training. bw = body weight; 1-RM = one repetition maximum; BOSU = BOth Sides Utilized; ROM = Range of Motion; ML = mediolateral.

	Intro-phase (1 week)	Block I (3 weeks)	Block II (3 weeks)	Block III (3 weeks)
	~2 × 12 reps (with low weights)	3 × 15 reps (50% of the 1-RM)	3–4 × 15 reps (60% of the 1-RM)	4 × 15 reps (60% of the 1-RM)
**I-FRT**				
Cross-Trainer	10 min	10 min	10 min	10 min
Squats	150° knee flex/ext angle on AIREX coordination rocker board round	120° knee flex/ext angle on Thera-Band balance pads placed on AIREX coordination rocker board angled	100° knee flex/ext angle on AIREX balance pad placed on AIREX coordination rocker board angled	100° knee flex/ext angle on BOSU ball or Variosensa board
Front lunges	Thera-Band Balance Pads (front foot)	AIREX coordination rocker board round (front foot) and Thera-Band Balance Pads (rear foot)	AIREX balance pad (front foot) and Thera-Band Balance Pads (rear foot)	AIREX balance pad (front foot) and AIREX balance spinner soft (rear foot)
Core Exercise (Bridge Exercise)	No additional device	TOGU DYNAIR (under feet)	TOGU DYNAIR (under shoulder) & BOSU (under feet)	Swiss ball (under feet)
Walking with dumbbells	2 min without dumbbells on terrasensa flats	3 min with 5% of bw on terrasensa flats	4 min with 10% of bw on terrasensa classics	5 min with 15% of bw on terrasensa
Walking with dumbbells	2 min without dumbbells	3 min with 5% of BW	4 min with 10% of BW	5 min with 15% of BW
**S-MRT**				
Cross-Trainer	10 min	10 min	10 min	10 min
Smith-Machine	150° knee flex/ext angle	120° knee flex/ext angle	100° knee flex/ext angle	100° knee flex/ext angle
Leg-Press	90° knee flex/ext angle	90° knee flex/ext angle	90° knee flex/ext angle	90° knee flex/ext angle
Core Exercise	Bridge exercise (2 × 15 reps)	Bridge exercise (3 × 20 reps)	Crunches (4 × 20 reps)	Air Bike Crunches (4 × 20 reps)
Walking with dumbbells	2 min without dumbbells	3 min with 5% of BW	4 min with 10% of BW	5 min with 15% of BW
**S-MRT**_**HIP**_				
Cross-Trainer	10 min	10 min	10 min	10 min
Adductor	Habituation	Full ROM	Full ROM	Full ROM
Abductor	Habituation	Full ROM	Full ROM	Full ROM
Adductor Thera-Band	Habituation	Full ROM	Full ROM	Full ROM
Abductor Thera-Band	Habituation	Full ROM	Full ROM	Full ROM
Core Exercise	Side plank on knees	Side Crunches	Standing Oblique Crunch	Russian Sitting twist with dumbbell 5% bw
treadmill walking on robowalk	2 min habituation	3 min with ML pull above knee joint with 5% of BW	4 min with ML pull at ankles with 5% of BW	5 min with ML pull above knee joint and at ankles with 5% of BW, respectively

#### Training intensity

The combined load (weight) for the two main exercises during the last training phase, transcribed from the participants’ training sheets. In addition, we assessed the mean Time under Tension (TuT) for the prescribed 15 repetitions.

### Data analysis

Prior to the main statistical analysis, normal distribution was checked by visual inspection and tested with the Kolmogorov-Smirnov test for each dependent variable. In addition, Levene’s test for homogeneity of variance was conducted. Baseline differences were tested between groups with a one-way ANOVA or a Kruskal-Wallis test depending on data distribution and homogeneity. We calculated pre-post differences for all variables of interest. To test our hypothesis, we ran several one-way analyses of variance with planned contrasts between ‘unstable and stable’ to test our first hypothesis and planned contrasts between the two stable groups to test our second hypothesis.

In addition, differences in the absolute training intensity within the last training block were analysed. Therefore, the absolute training load, defined as the added weight of the two main exercises, for each group and the TuT of the main exercises were investigated. We used pre-planned independent two-sided *t*-tests (or non-parametrical alternatives) to investigate differences between groups. Ryan-Holm-Bonferroni^64^ corrected *p*-values for the *t-*tests are reported.

To improve readability, we calculated the effect size Cohen’s *d* for ANOVAs. Exploratory Software for Confidence Intervals was used for the calculation of Cohen’s *d*_*unb*_ (an unbiased estimate of the population effect size *δ*), associated 95% confidence intervals (see Cumming 2012 for details)^65^ for planned contrasts and *t*-tests. Following Cohen (1988)^66^, *d*-values ≤ 0.49 indicate small effects, 0.50 ≤ *d* ≤ 0.79 indicate medium effects, and *d* ≥ 0.80 indicate large effects. However, given that we did not implement a passive control group, even small effect sizes can be regarded as meaningful. Alpha level was set at 5%. The effect size serves as a measure of how much the results deviate from the null hypothesis^65–67^. For the other tests we used SPSS version 26.0 (SPSS Inc., Chicago, IL, USA).

## Supplementary information

Supplementary Dataset 1.

## Data Availability

All data analysed during this study are included in this published article (and its Supplementary Information files).
